# Artificial Intelligence for the Prediction of Helicobacter Pylori Infection in Endoscopic Images: Systematic Review and Meta-Analysis Of Diagnostic Test Accuracy

**DOI:** 10.2196/21983

**Published:** 2020-09-16

**Authors:** Chang Seok Bang, Jae Jun Lee, Gwang Ho Baik

**Affiliations:** 1 Department of Internal Medicine Hallym University College of Medicine Chuncheon Republic of Korea; 2 Institute for Liver and Digestive Diseases Hallym University Chuncheon Republic of Korea; 3 Institute of New Frontier Research Hallym University College of Medicine Chuncheon Republic of Korea; 4 Division of Big Data and Artificial Intelligence Chuncheon Sacred Heart Hospital Chuncheon Republic of Korea; 5 Department of Anesthesiology and Pain Medicine Hallym University College of Medicine Chuncheon Republic of Korea

**Keywords:** artificial intelligence, convolutional neural network, deep learning, machine learning, endoscopy, Helicobacter pylori

## Abstract

**Background:**

*Helicobacter pylori* plays a central role in the development of gastric cancer, and prediction of *H pylori* infection by visual inspection of the gastric mucosa is an important function of endoscopy. However, there are currently no established methods of optical diagnosis of *H pylori* infection using endoscopic images. Definitive diagnosis requires endoscopic biopsy. Artificial intelligence (AI) has been increasingly adopted in clinical practice, especially for image recognition and classification.

**Objective:**

This study aimed to evaluate the diagnostic test accuracy of AI for the prediction of *H pylori* infection using endoscopic images.

**Methods:**

Two independent evaluators searched core databases. The inclusion criteria included studies with endoscopic images of *H pylori* infection and with application of AI for the prediction of *H pylori* infection presenting diagnostic performance. Systematic review and diagnostic test accuracy meta-analysis were performed.

**Results:**

Ultimately, 8 studies were identified. Pooled sensitivity, specificity, diagnostic odds ratio, and area under the curve of AI for the prediction of *H pylori* infection were 0.87 (95% CI 0.72-0.94), 0.86 (95% CI 0.77-0.92), 40 (95% CI 15-112), and 0.92 (95% CI 0.90-0.94), respectively, in the 1719 patients (385 patients with *H pylori* infection vs 1334 controls). Meta-regression showed methodological quality and included the number of patients in each study for the purpose of heterogeneity. There was no evidence of publication bias. The accuracy of the AI algorithm reached 82% for discrimination between noninfected images and posteradication images.

**Conclusions:**

An AI algorithm is a reliable tool for endoscopic diagnosis of *H pylori* infection. The limitations of lacking external validation performance and being conducted only in Asia should be overcome.

**Trial Registration:**

PROSPERO CRD42020175957; https://www.crd.york.ac.uk/prospero/display_record.php?RecordID=175957

## Introduction

More than half of the world’s population is infected with the *Helicobacter pylori* bacteria [1], which is associated with various disorders, such as gastritis, peptic ulcer, mucosa-associated lymphoid tissue lymphoma, gastric adenocarcinoma, and immune thrombocytopenic purpura [2,3]. The infection causes chronic atrophic gastritis, intestinal metaplasia, dysplasia, and gastric cancer in sequence [4]. The International Agency for Research on Cancer has categorized *H pylori* as a group 1 carcinogen [5]. Elimination of this pathogen is considered the most promising strategy for the prevention of gastric cancer [6,7].

An important aspect of endoscopy is the ability to predict *H pylori*–induced gastritis by visual inspection of the gastric mucosa to identify patients at high risk for gastric cancer. Representative features of *H pylori*–induced gastritis have been reported in the literature, including mucosal edema, atrophy, diffuse erythema, enlargement of mucosal folds, or mucosal nodularity [8,9]. The regular arrangement of collecting venules and fundic gland polyps has been suggested as a predictive marker of the *H pylori*–naïve stomach. Also, map-like redness under white-light imaging (WLI) or a cracked pattern under blue-laser imaging (BLI) have been suggested as features of a posteradicated gastric mucosa [8,9].

These endoscopic features do not have objective indicators, and there is the potential for interobserver or intraobserver variability in the optical diagnosis of *H pylori*–infected mucosa [10]. Although expert endoscopists might reliably identify an *H pylori* infection with meticulous visual inspection of the mucosa during endoscopic examination, novice endoscopists require substantial time to perform this task efficiently. Image-enhanced endoscopy (IEE), such as narrow-band imaging (NBI), BLI, or linked color imaging (LCI), with or without magnification, has been developed. Previous studies have indicated increased diagnostic accuracy of gastrointestinal neoplasms with the application of these modalities during endoscopic examination [11,12]. This also requires considerable training and prolonged procedure time. There are no uniform features of *H pylori* infection in IEE [12]. Therefore, there are currently no established methods of optical endoscopic diagnosis of *H pylori* infection. Definitive diagnosis continues to require endoscopic biopsy, which is categorized as an invasive diagnostic test.

Artificial intelligence (AI) has been increasingly adopted in clinical practice, especially for image recognition and classification [13]. This technique has shown promising diagnostic performance using endoscopic images, such as detecting cancer or neoplastic lesions and classifying neoplastic or nonneoplastic lesions in the gastrointestinal tract [14]. Application of AI in endoscopic examination is expected to be useful. It can help detect *H pylori* infection in real time and determine the optimum definitive test for *H pylori* infection. There has been no diagnostic test accuracy meta-analysis of AI for the prediction of *H pylori* infection using endoscopic images.

This study aimed to evaluate the diagnostic performance of AI for the diagnosis of *H pylori* infection using endoscopic images.

## Methods

### Ethics

This study adhered to the guidelines of the Preferred Reporting Items for a Systematic Review and Meta-analysis of Diagnostic Test Accuracy Studies (PRISMA-DTA) [15]. The protocol of this study was registered at the International Prospective Register of Systematic Reviews (PROSPERO) [CRD42020175957] on March 2019 before initiating the study. Approval of the institutional review board was exempted as only anonymized data was collected from the literature.

### Literature Searching Strategy

Two independent evaluators (CSB and JJL) having published 23 systematic reviews and 11 PROSPERO protocols searched PubMed, Embase, and the Cochrane Library using common keywords relevant to *H pylori* infection and AI (inception to March 2020). The abstracts of all identified studies were reviewed to exclude irrelevant articles. Full-text reviews were conducted to determine whether the inclusion criteria were satisfied in all the studies. Bibliographies were also reviewed to identify additional relevant articles. Disagreements between the evaluators were resolved by consultation with a third evaluator (GHB). The details are presented in Multimedia Appendix 1.

### Selection Criteria

We included studies that met the following criteria: (1) studies with endoscopic images of *H pylori* infection as a case group and endoscopic images without *H pylori* infection as a negative control group; (2) application of the AI algorithm for the prediction of *H pylori* infection; (3) inclusion of diagnostic performance indices of the AI algorithm, including sensitivity, specificity, positive predictive value (PPV), negative predictive value (NPV), positive likelihood ratio (PLR), negative likelihood ratio (NLR), diagnostic odds ratio (DOR), or accuracy, which enable an estimation of true positive (TP), false positive (FP), false negative (FN), and true negative (TN) values for the prediction of *H pylori* infection using endoscopic images; (4) prospective or retrospective study design; (5) human adult subjects; and (6) full-text publications written in English. The exclusion criteria included (1) narrative reviews; (2) letters, comments, editorials, or protocol studies; (3) guidelines; and (4) systematic reviews and meta-analyses. Studies meeting at least one of the exclusion criteria were excluded from the analysis.

### Methodological Quality

The Quality Assessment of Diagnostic Accuracy Studies–2 (QUADAS-2) tool was used to determine the methodological quality of the included articles. This tool contains 4 domains: patient selection, index test, reference standard, and flow and timing [16]. Each domain was assessed in terms of high, low, or unclear risk of bias, and the first 3 domains were also assessed in terms of high, low, or unclear concerns regarding applicability [16]. Review Manager version 5.3.3 (RevMan for Windows 7, Nordic Cochrane Centre) was used to generate the summary figure of the methodological quality evaluation. Data extraction, primary and modifier-based analyses, and statistical analysis are described in Multimedia Appendix 2 [17-20].

## Results

### Identification of Relevant Studies

**Figure 1:**
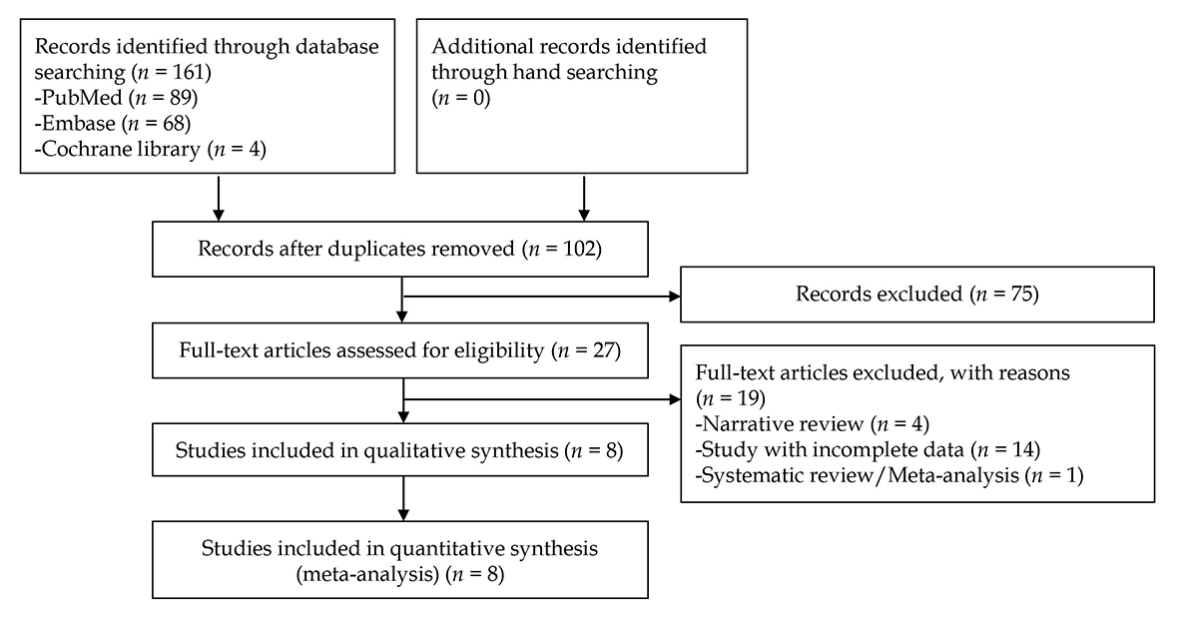
Flow diagram of the identification of relevant studies.

In total, 161 articles were identified by searching 3 electronic databases. Among them, 59 were duplicate studies, and 75 were excluded during the initial screening by reviewing titles and abstracts. Full texts of the remaining 27 articles were thoroughly reviewed. Among these, 19 studies were excluded from the final analysis due to the following reasons: narrative review (n=4), incomplete data (n=14), and systematic review or meta-analysis (n=1; the topic of this systematic review was the role of nonmagnified endoscopy for the assessment of *H pylori* infection) [8]. The remaining 8 studies [9,10,21-26] were included in the final analysis. Figure 1 illustrates a flow diagram showing the process used to identify the relevant articles.

### Characteristics of the Included Studies

The included studies could be categorized by analysis based on the number of enrolled patients [9,10,22,23,25,26] and number of enrolled images [9,10,21,24]. Two studies [9,10] presented both patient-based and image-based analyses. Enrolled studies presented performance of the AI algorithm with test dataset (internal validation), and there was no study that presented external validation performance.

Among the 8 studies [9,10,21-26] included for the prediction of *H pylori* infection using endoscopic images, we identified 1719 patients (385 patients with *H pylori* infection vs 1334 controls). Additionally, 2855 endoscopic images with *H pylori* infection and 2287 control images including 514 posteradicated images were identified.

Among the studies, 5 were retrospectively conducted [9,10,21,22,25], and 3 [23,24,26] were prospectively conducted. All studies were conducted in Asia, and the age of the enrolled population ranged from a mean of 48.6 years to a median of 64 years. Most studies [9,21-24,26] established the AI algorithm based on the convolutional neural network (CNN), whereas 2 studies [10,25] established support vector machine (SVM)-based algorithms. Most studies [9,21,22,24-26] used endoscopic images with WLI, whereas a study by Yasuda et al [10] used endoscopic images with LCI, and Nakashima et al [23] used LCI and BLI images in addition to endoscopic images with WLI. While most studies [9,10,21,22,24-26] presented the performance of the AI algorithm as a single primary outcome, one study [23] also presented a feature map, which implies visualizing where established AI algorithms pay attention to and indicate a region of interest.

These characteristics (modifiers) were evaluated as potential sources of heterogeneity through the subgroup analysis and meta-regression. Detailed characteristics of the studies are presented in Table 1.

**Table 1:** Clinical characteristics of the included studies.

Study, format, nationality	Type of AI^a^	Type of endoscopy, diagnostic method of *Helicobacter pylori* infection	Number of cases in test dataset	Number of controls in test dataset	Age of patients in test dataset; gender in patients in test dataset (M/F^b^)	TP^c^	FP^d^	FN^e^	TN^f^	Unit of analysis
Yasuda et al [10], retrospective, Japan	Support vector machine	LCI^g^; more than 2 different tests in each case (histology, serum antibody, stool antigen, urea breath test)	42 *H pylori* patients	63 controls (46 posteradication patients and 17 uninfected patients)	Median 64 years (range 26-88); (61/44)	38	9	4	54	Patient-based
—	—	—	210 *H pylori*–positive images	315 control images (230 posteradication and 85 uninfected images)	—	161	70	49	245	Image-based
—	—	—	210 *H pylori*–positive images	85 uninfected images (*H pylori*–naïve)	—	161	9	49	76	Image-based (infected vs uninfected)
—	—	—	210 *H pylori*–positive images	230 posteradication images	—	161	61	49	169	Image-based (infected vs after-eradication)
—	—	—	85 uninfected images	230 posteradication images	—	76	61	9	169	Image-based (uninfected vs after-eradication)
Zheng et al [21], retrospective, China	CNN^h^	WLI^i^; histology with immunohistochemistry (if negative, urea breath test was done)	2575 *H pylori*–positive images	1180 control images (whether posteradication or uninfected images is unknown)	Mean 48.6 years (SD 12.9); (220/232)	2359	17	216	1163	Image-based
Shichijo et al [9], retrospective, Japan	CNN	WLI; serum or urine antibody, stool antigen, urea breath test	70 *H pylori*–positive patients	777 controls (284 posteradication and 493 uninfected images)	—	44	47	26	730	Patient-based
—	—	—	59 *H pylori*–positive images	477 uninfected images (*H pylori*–naïve)	—	44	12	15	465	Image-based (infected vs uninfected)
—	—	—	55 *H pylori*–positive images	182 posteradication images	—	44	35	11	147	Image-based (infected vs after-eradication)
—	—	—	481 uninfected images	249 posteradication images	—	465	102	16	147	Image-based (uninfected vs after-eradication)
Nakashima et al [23], prospective, Japan	CNN	WLI; serum antibody (*H pylori* IgG ≥10 U/mL was considered positive)	30 *H pylori* patients	30 controls (uninfected patients; *H pylori*–naïve)	—	20	12	10	18	Patient-based
—	—	LCI	—	—	—	29	1	1	29	Patient-based
—	—	BLI^j^-bright	—	—	—	29	4	1	26	Patient-based
Itoh et al [24], prospective, Japan	CNN	WLI; serum antibody (*H pylori* IgG ≥10 U/mL was considered positive)	15 *H pylori*–positive images	15 control images (uninfected patients; *H pylori*–naïve)	—	13	2	2	13	Image-based
Shichijo et al [22], retrospective, Japan	CNN	WLI; serum or urine antibody, stool antigen, urea breath test	72 *H pylori* patients	325 controls (uninfected patients; *H pylori*–naïve)	mean 50.4 (SD 11.2), (168/226)	64	41	8	284	Patient-based
Huang et al [25], retrospective, Taiwan	Sequential forward floating selection with SVM^k^	WLI; histology (3 pairs of samples from the topographic sites, including antrum, body, and cardia were obtained in a uniform way)	130 *H pylori* patients	106 controls (whether posteradication or uninfected patients is unknown)	—	128	21	2	85	Patient-based
Huang et al [26], prospective, Taiwan	Refined feature selection with neural network	WLI; histology (3 pairs of samples from the topographic sites, including antrum, body, and cardia were obtained in a uniform way)	41 *H pylori* patients	33 controls (whether posteradication or uninfected patients is unknown)	—	35	3	6	30	Patient-based

^a^AI: artificial intelligence.

^b^M/F: make/female.

^c^TP: true positive.

^d^FP: false positive.

^e^FN: false negative.

^f^TN: true negative.

^g^LCI: linked color imaging.

^h^CNN: convolutional neural network.

^i^WLI: white-light imaging.

^j^BLI: blue-laser imaging.

^k^SVM: support vector machine.

### Methodological Quality of the Studies

Among the 8 studies [9,10,21-26] in the final analysis, 6 studies [9,10,21,22,25,26] showed low risk of bias, and 2 studies [23,24] showed high risk of bias in patient selection.

In terms of the patient selection, 4 studies [9,10,21,22] used multiple tests, including a biopsy, serology (serum anti–*H pylori* IgG titer), stool antigen test, urine examination (urine anti–*H pylori* IgG titer), or a urea breath test for the determination of *H pylori* infection. Two studies [25,26] used only gastric biopsy; however, 3 pairs of samples from the topographic sites, including the antrum, body, and cardia were obtained in a uniform way. The remaining 2 studies [23,24] used only serology (serum anti–*H pylori* IgG titer) for the determination of *H pylori* infection. Although a serology test is convenient and widely used in Japan, local validation is essential to determine the best cutoff values. A recent Cochrane review suggested that serology is less accurate for the diagnosis of *H pylori* infection compared with the urea breath test [27].

For concerns regarding image selection, most studies [9,10,21,22,25,26] did not limit the specific topographic area of the endoscopic still images for enrollment in the study. However, 2 studies [23,24] used still images limited to the lesser curvature of the stomach. Considering that topographic distribution and density of *H pylori* is different according to the stage of gastritis, the results of these studies may include a risk of bias.

Considering the commonly detected pitfalls in patient and image selection described above, these 2 studies [23,24] were rated as high risk in the patient selection domain in the risk of bias evaluation.

**Figure 2:**
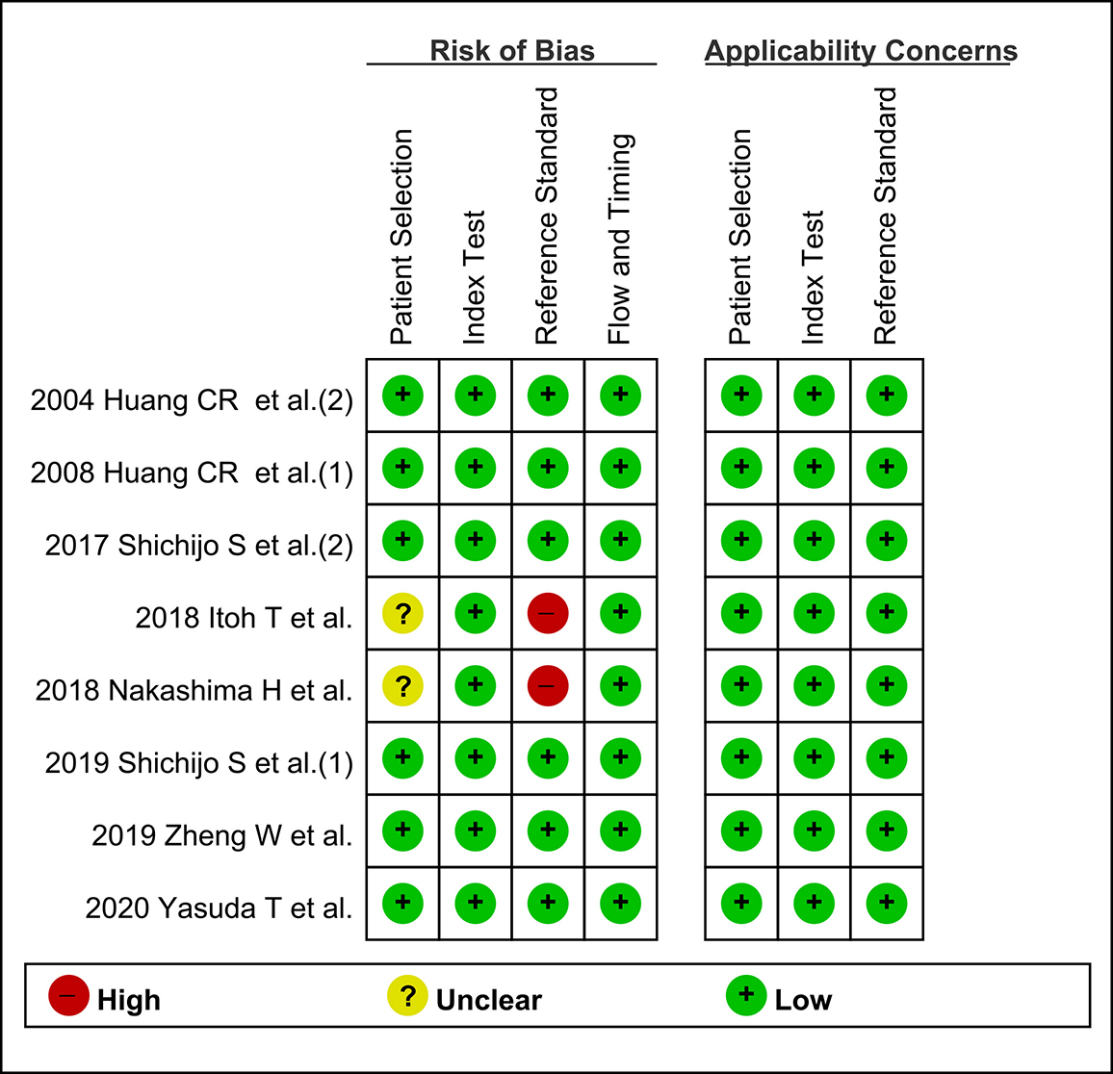
Quality Assessment of Diagnostic Accuracy Studies–2 for the assessment of the methodological qualities of all the enrolled studies. (+) denotes low risk of bias, (?) denotes unclear risk of bias, (-) denotes high risk of bias.

Overall, studies [23,24] with high risk in at least 1 of the 7 domains were rated as low methodological quality in the subgroup analysis (Figure 2).

### Diagnostic Test Accuracy of Artificial Intelligence for the Prediction of Helicobacter pylori Infection

**Figure 3:**
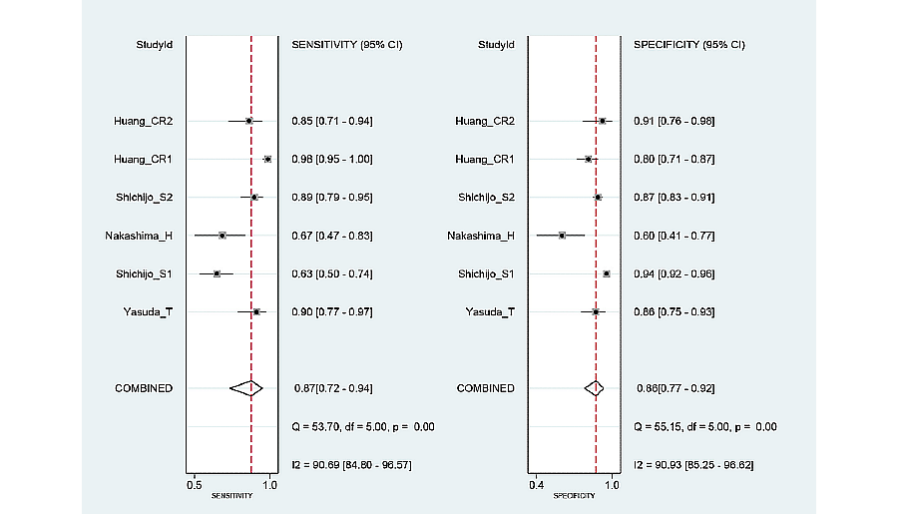
Forest plots of sensitivity and specificity of artificial intelligence algorithm for the prediction of Helicobacter pylori infection in endoscopic images.

**Figure 4:**
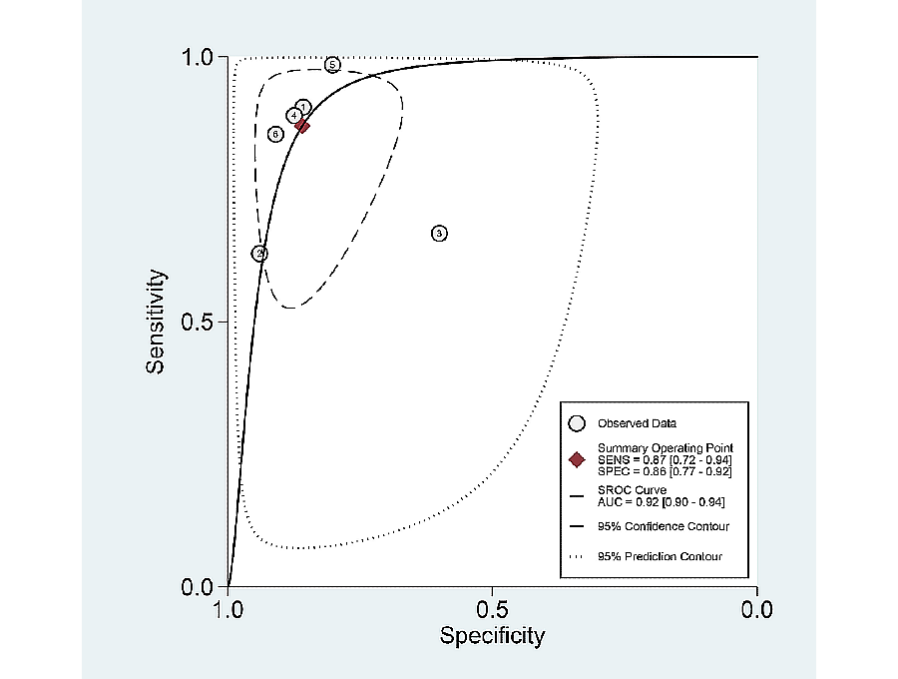
Summary receiver operating characteristic curve with 95% confidence region and prediction region for the prediction of Helicobacter pylori infection in endoscopic images.

**Figure 5:**
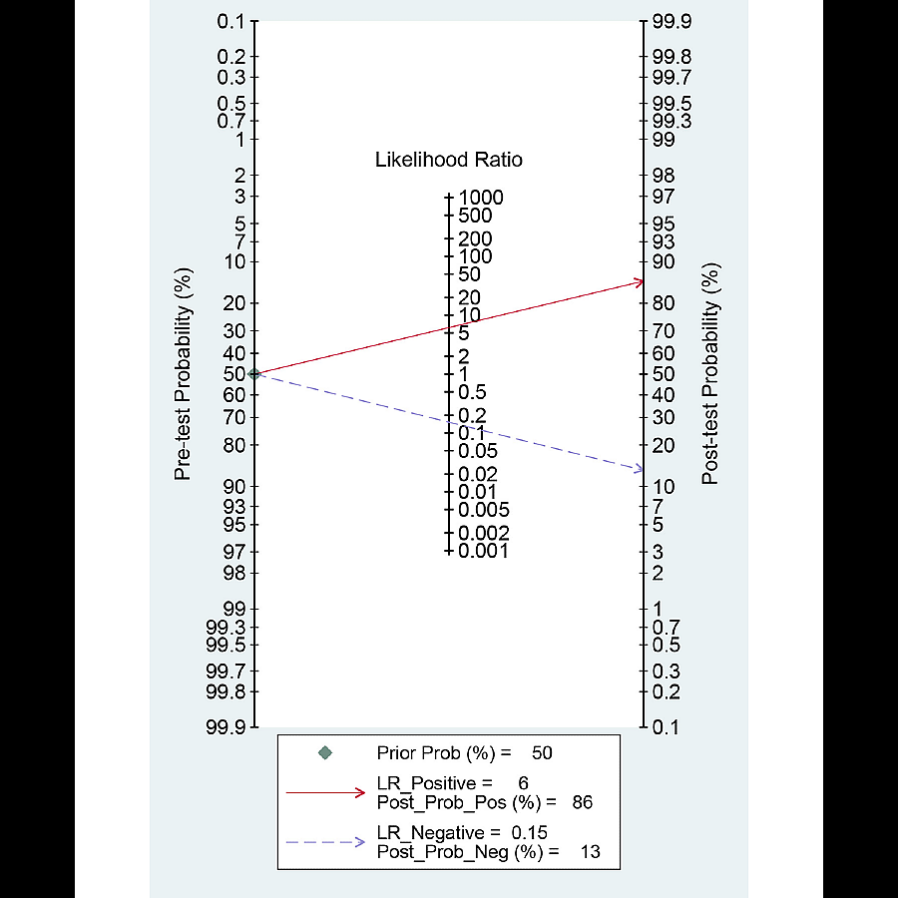
Fagan normogram for the prediction of Helicobacter pylori infection in endoscopic images.

Among the 6 studies [9,10,22,23,25,26] of patient-based analysis, the sensitivity, specificity, PLR, NLR, DOR, and area under the curve (AUC) with 95% CI of AI for the prediction of *H pylori* infection were 0.87 (95% CI 0.72-0.94), 0.86 (95% CI 0.77-0.92), 6.2 (95% CI 3.8-10.1), 0.15 (95% CI 0.07-0.34), 40 (95% CI 15-112), and 0.92 (95% CI 0.90-0.94), respectively (Table 2, Figure 3). The SROC curve, with a 95% confidence region and prediction region, is illustrated in Figure 4. To investigate the clinical utility of AI, a Fagan nomogram was generated. Assuming 50% prevalence of *H pylori* infection, the Fagan nomogram shows that the posterior probability of *H pylori* infection was 86% if the test was positive, and the posterior probability of absence of *H pylori* infection was 13% if the test was negative (Figure 5).

**Table 2:** Summary of diagnostic test accuracy and subgroup analysis of the included studies with patient-based analysis.

Subgroup		Number of included studies	Sensitivity (95% CI)	Specificity (95% CI)	PLR^a^	NLR^b^	DOR^c^	AUC^d^
Value of meta-analysis in all included studies		6	0.87 (0.72-0.94)	0.86 (0.77-0.92)	6.2 (3.8-10.1)	0.15 (0.07-0.34)	40 (15-112)	0.92 (0.90-0.94)
**Methodological quality of included studies^e^**								
	High quality	5	0.89 (0.75-0.96)	0.88 (0.83-0.92)	7.7 (5.6-10.6)	0.12 (0.05-0.28)	64 (32-129)	0.94 (0.91-0.95)
	Low quality	1	Null	Null	Null	Null	Null	Null
**Total number of included patients^e^**								
	≤100	4	0.90 (0.73-0.97)	0.88 (0.81-0.93)	7.6 (5.3-10.9)	0.11 (0.04-0.32)	68 (29-158)	0.94 (0.91-0.95)
	<100	2	Null	Null	Null	Null	Null	Null
**Format of study**								
	Retrospective	4	0.90 (0.73-0.97)	0.88 (0.81-0.93)	7.6 (5.3-10.9)	0.11 (0.04-0.32)	68 (29-158)	0.94 (0.91-0.95)
	Prospective	2	Null	Null	Null	Null	Null	Null
**Published year**								
	After 2010	4	0.80 (0.64-0.90)	0.86 (0.73-0.93)	5.6 (2.8-11.3)	0.24 (0.13-0.45)	23 (8-72)	0.90 (0.87-0.92)
	Before 2010	2	Null	Null	Null	Null	Null	Null
**Type of AI^f^**								
	Neural network–based	4	0.78 (0.64-0.87)	0.87 (0.74-0.94)	6.0 (2.7-13.0)	0.26 (0.15-0.44)	23 (7-73)	0.89 (0.86-0.91)
	SVM^g^-based	2	Null	Null	Null	Null	Null	Null
**Type of endoscopic image**								
	WLI^h^	5	0.86 (0.67-0.95)	0.86 (0.75-0.92)	6.1 (3.4-10.9)	0.16 (0.06-0.42)	37 (11-124)	0.92 (0.89-0.94)
	LCI^i^	1	Null	Null	Null	Null	Null	Null
Classifying performance between *Helicobacter pylori*–positive vs *H pylori*–naïve patients		2	0.82 (0.74-0.89)	0.85 (0.81-0.89)	3.5 (0.8-14.3)	0.27 (0.05-1.41)	13 (0.8-229)	Null

^a^PLR: positive likelihood ratio.

^b^NLR: negative likelihood ratio.

^c^DOR: diagnostic odds ratio.

^d^AUC: area under the curve.

^e^These modifiers were significant in the meta-regression analysis.

**^f^**AI: artificial intelligence.

**^g^**SVM: support vector machine.

**^h^**WLI: white-light imaging.

**^i^**LCI: linked color imaging.

Among the 4 studies [9,10,21,24] of image-based analysis, sensitivity, specificity, PLR, NLR, DOR, and AUC with 95% CI of AI for the prediction of *H pylori* infection were 0.81 (95% CI 0.68-0.90), 0.93 (95% CI 0.82-0.98), 12.3 (95% CI 3.8-39.2), 0.20 (95% CI 0.11-0.38), 61 (95% CI 11-322), and 0.93 (95% CI 0.90-0.95), respectively (Table 3).

Only 2 studies [9,10] reported outcomes related to discrimination between noninfected images and posteradication images. Therefore, a meta-analysis was not possible. Pooled analysis of the crude value of TP, FP, FN, and TN revealed that accuracy of the AI algorithm reached 82.01% (857/1045).

Additionally, only 2 studies [9,10] reported outcomes regarding discrimination between images showing *H pylori* infection and posteradication images. Therefore, a meta-analysis was not possible. However, pooled analysis of the crude value of TP, FP, FN, and TN revealed that accuracy of the AI algorithm reached 77.0% (521/677).

Regarding comparison of the performance between AI and endoscopists, only 2 studies presented outcomes [10,22]. In the study by Yasuda et al [10], the diagnostic accuracy of an SVM-based AI algorithm was superior to that of inexperienced endoscopists. However, there was no significant difference between experienced endoscopists and the AI algorithm [10]. The accuracy of a CNN-based AI algorithm reached 87.7% in the study by Shichijo et al [22], while the accuracy achieved by endoscopists was 82.4%. The difference was statistically significant between the AI algorithm and endoscopists (5.3%, 95% CI 0.3-10.2) [22].

### Exploring Heterogeneity With Meta-Regression and Subgroup Analysis

**Figure 6:**
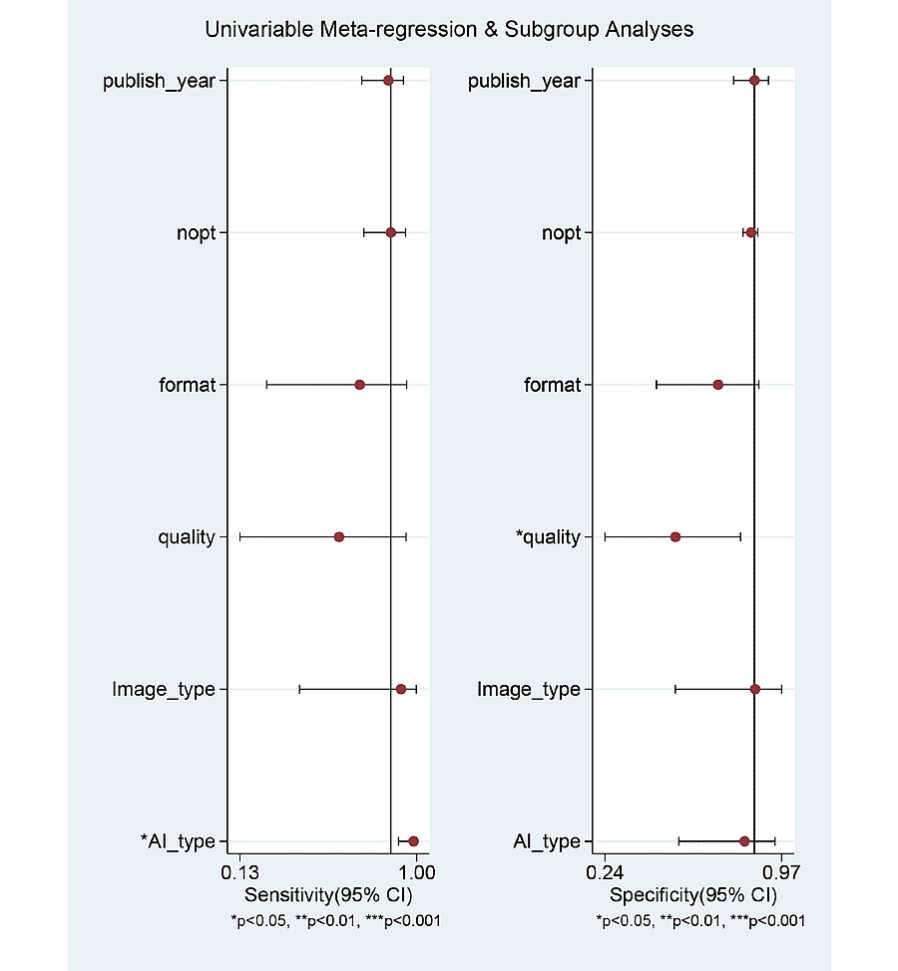
Meta-regression for the reason of heterogeneity in the diagnostic test accuracy meta-analysis. nopt: number of patients.

For the prediction of *H pylori* infection using endoscopic images, the SROC curve was generated in the patient-based studies. The shape of the curve was symmetric (Figure 4). We observed a negative correlation coefficient between logit transformed sensitivity and specificity (–0.22) and an asymmetric parameter, β, with a nonsignificant *P* value (*P*=.29) indicating no heterogeneity among the studies. However, the 95% prediction region in the SROC curve was wide, and the methodological quality among the included studies (*P*<.001) and total number of included patients (*P*=.03) were found to be the source of heterogeneity in the joint model of meta-regression (published year [*P*=.41], study format [*P*=.10], type of endoscopic image [*P*=.92], and type of AI [*P*=.07]; Figure 6). Subgroup analyses, based on the modifiers of heterogeneity, showed higher AUCs or DORs in studies with a large population of patients (≤100) or those demonstrating high methodological quality (Table 2).

In terms of the image-based analysis, the overall number of included studies was 4, and subgroup analysis was possible with only 3 studies. Studies with CNN (vs SVM) and studies with WLI (vs LCI) showed higher AUCs or DORs (Table 3). However, these modifiers (type of AI and type of endoscopic imaging) were not a significant covariate in the meta-regression analysis (total number of included patients [*P*=.06], methodological quality [*P*=.68], published year [*P*=.78], study format [*P*=.68], type of endoscopic image [*P*=.72], or type of AI [*P*=.72]).

The enrolled studies included various types of control groups. The fundamental question of this study was whether the AI algorithm could differentiate endoscopic images between an *H pylori*–positive and a naïve gastric mucosa. Table 1 shows the types of control group included in each study. Two studies clearly presented the classifying performance of an AI algorithm discriminating *H pylori*–positive and *H pylori*–naïve in a patient-based analysis, and there were 3 with image-based analysis. Subgroup analysis was also performed and showed slightly lower AUCs or DORs in patient-based or image-based analysis (Table 2 and 3). However, this factor (studies with clearly presented classifying performance data discriminating *H pylori*–positive and *H pylori*–naïve group) was not a significant modifier in the meta-regression analysis (*P*=.21 in the patient-based analysis, and *P*=.10 in the image-based analysis).

**Table 3:** Summary of diagnostic test accuracy and subgroup analysis of the included studies with image-based analysis.

Subgroup			Number of included studies		Sensitivity (95% CI)		Specificity (95% CI)		PLR^a^		NLR^b^		DOR^c^		AUC^d^
Value of meta-analysis in all the included (bivariate and HSROC^e^ method)			4		0.81 (0.68-0.90)		0.93 (0.82-0.98)		12.3 (3.8-39.2)		0.20 (0.11-0.38)		61 (11-322)		0.93 (0.90-0.95)
Value of meta-analysis in all the included (Moses-Shapiro-Littenberg method)					0.90 (0.89-0.91)		0.94 (0.93-0.95)		11.1 (1.6-76.2)		0.20 (0.08-0.52)		56 (5-591)		0.90 (0.71-0.99)
**Methodological quality of included studies**															
	High quality	3		0.90 (0.87-0.91)		0.94 (0.93-0.95)		13.1 (1.4-124.5)		0.22 (0.08-0.62)		61 (4-919)		0.87 (0.43-0.99)	
	Low quality	1		Null		Null		Null		Null		Null		Null	
**Total number of included patients**															
	≤100	3		0.90 (0.87-0.91)		0.94 (0.93-0.95)		13.1 (1.4-124.5)		0.22 (0.08-0.62)		61 (4-919)		0.87 (0.43-0.99)	
	<100	1		Null		Null		Null		Null		Null		Null	
**Format of study**															
	Retrospective	3		0.90 (0.87-0.91)		0.94 (0.93-0.95)		13.1 (1.4-124.5)		0.22 (0.08-0.62)		61 (4-919)		0.87 (0.43-0.99)	
	Prospective	1		Null		Null		Null		Null		Null		Null	
**Published year**															
	After 2010	4		0.90 (0.89-0.91)		0.94 (0.93-0.95)		11.1 (1.6-76.2)		0.20 (0.08-0.52)		56 (5-591)		0.90 (0.71-0.99)	
	Before 2010	0													
**Type of AI^f^**															
	Neural network–based	3		0.91 (0.90-0.92)		0.97 (0.96-0.97)		16.8 (2.0-141.7)		0.17 (0.05-0.61)		98 (6-1640)		0.95 (0.75-0.99)	
	SVM^g^–based	1		Null		Null		Null		Null		Null		Null	
**Type of endoscopic image**															
	WLI^h^	3		0.91 (0.90-0.92)		0.97 (0.96-0.97)		16.8 (2.0-141.7)		0.17 (0.05-0.61)		98 (6-1640)		0.95 (0.75-0.99)	
	LCI^i^	1		Null		Null		Null		Null		Null		Null	
Classifying performance between *Helicobacter pylori*–positive vs *H pylori*–naïve images			3		0.77 (0.71-0.82)		0.96 (0.94-0.98)		11.8 (3.7-38.3)		0.26 (0.21-0.32)		53 (17-161)		0.88 (0.79-0.96)

^a^PLR: positive likelihood ratio.

^b^NLR: negative likelihood ratio.

^c^DOR: diagnostic odds ratio.

^d^AUC: area under the curve.

^e^HSROC: hierarchical summary receiver operating characteristic.

^f^AI: artificial intelligence.

^g^SVM: support vector machine.

^h^WLI: white-light imaging.

^i^LCI: linked color imaging.

### Publication Bias

**Figure 7:**
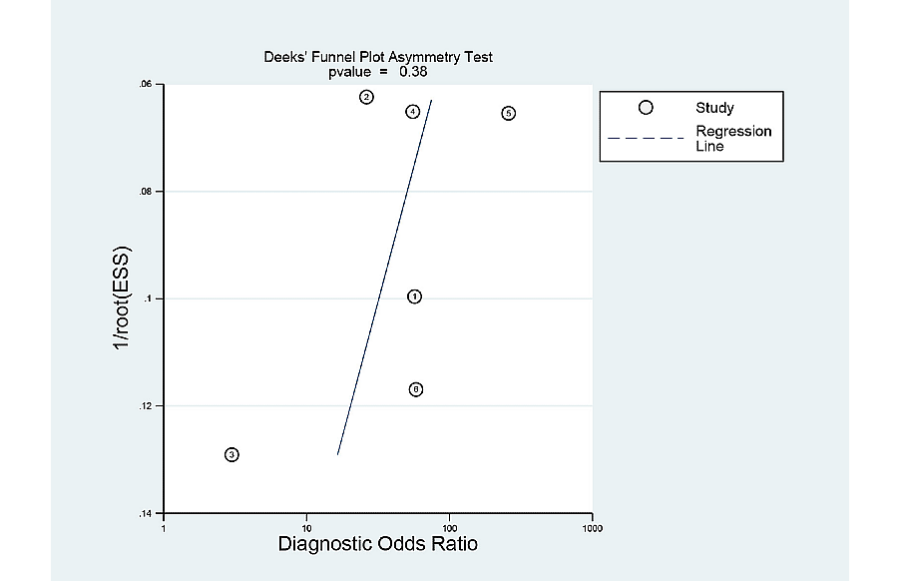
Deek funnel plot for the studies of patient-based analysis.

**Figure 8:**
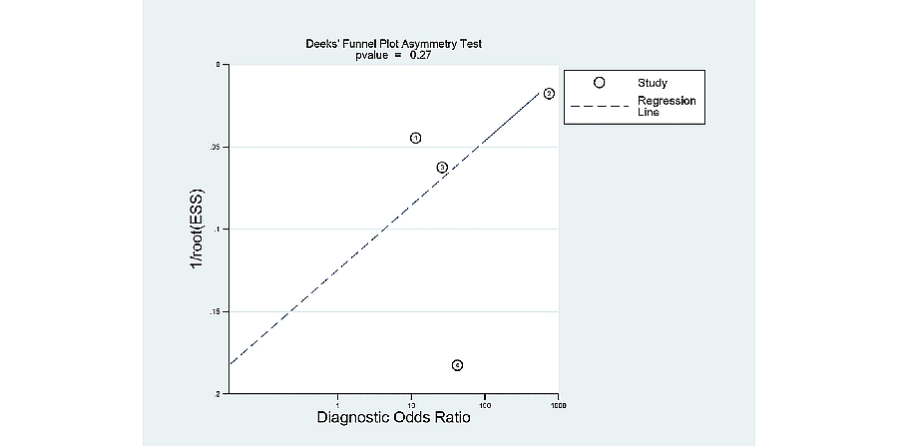
Deek funnel plot for the studies of image-based analysis.

Figure 7 shows the Deek funnel plot of studies of patient-based analysis and Figure 8 shows the Deek funnel plot of studies of image-based analysis. The plot was grossly symmetrical with respect to the regression line. The Deek funnel plot asymmetry test showed no evidence of publication bias (*P*=.38 in the patient-based analysis, and *P*=.27 in the image-based analysis).

## Discussion

### Principal Findings

This study presented the good performance of the AI algorithm applied to endoscopic diagnosis of *H pylori* infection, indicating that AI-assisted endoscopy is feasible in clinical practice. Indeed, this approach might be characterized as a computer-aided diagnosis, and the most important benefit consists of the improvement in diagnostic accuracy of conventional endoscopy with WLI [28]. Optical endoscopic diagnosis has operator-dependent characteristics, and the diagnostic process is completely subjective. However, AI-assisted endoscopy could be helpful in providing a second opinion and may help avoid operator dependency in diagnostic endoscopy [28]. Currently, it is unclear how endoscopists would react to a diagnosis made using AI (examples from the literature include approval, a learning opportunity, or “presenting an indolent attitude”) [28,29]. Therefore, a prospective study based on the application of AI in clinical practice (more specifically, in diagnostic endoscopy) is essential [30,31]. However, providing robust answers using an AI algorithm irrespective of the endoscopists’ inspection would be helpful to increase the likelihood of identifying important findings in diagnostic endoscopy. As endoscopic biopsy is an invasive procedure, application of a highly accurate AI algorithm in endoscopic examination may reduce the need for unnecessary biopsies in a substantial proportion of patients.

Another important finding of this study is the robustness of the diagnostic performance of the AI algorithm, irrespective of the modifiers detected during the systematic review process. Although studies based on a large population of patients presenting high methodological quality demonstrated higher diagnostic performance, this difference in diagnostic performance was not substantial. Neither the type of AI, such as CNN or SVM, nor the type of endoscopic images used, such as WLI, LCI, or BLI, affected overall diagnostic performance. Studies with patient-based analysis and image-based analysis commonly presented a good performance of AI for the diagnosis of *H pylori* infection (Tables 2 and 3).

AI is generally characterized as being of a black-box nature due to the difficulty in explaining the determination of the AI algorithm. The class activation map is a technique for visualizing the locations to which established AI algorithms pay attention and indicating a region of interest. This technique offers the possibility of explaining the determination of the AI algorithm. Although only one study [23] included in this systematic review adopted this type of feature map with the AI algorithm, this technique has now been widely adopted for the establishment of the AI algorithm and could be useful for the work of endoscopists, specifically for targeted biopsy in *H pylori* detection.

In terms of the IEE, the ultimate goal of this technique would be optical biopsy replacing invasive histologic examination with the aid of discrete differentiation and enhancement of surface mucosal features. Previous studies on the diagnosis of *H pylori* infection with WLI showed low sensitivity and poor interobserver agreement [11,32-34]. However, studies with IEE commonly showed increased diagnostic accuracy of premalignant or malignant lesions during endoscopic examination [11,12]. Previous studies with IEE also indicated the usefulness of LCI for the diagnosis of *H pylori* infection [35,36]. Although a recently published systematic review concluded that currently no established uniform findings exist for optical endoscopic diagnosis of *H pylori* infection [8], IEE continues to have potential for the differentiation of *H pylori* infection. The development of standardized validated indicators is required. The additive effect of magnifying endoscopy in NBI also showed promising results for the diagnosis of *H pylori* infection [37,38]. Due to insufficient data on IEE for the application of AI in this study, the real value of IEE with AI could not be evaluated. Further studies using various types of IEE with AI applications is essential.

### Limitations

Although, this review rigorously investigated the diagnostic accuracy of the AI algorithm for *H pylori* infection in endoscopic images, our analysis has several inevitable limitations originating from potential bias in each study. First, the diagnostic performance of AI could have been exaggerated. It is more likely that the endoscopic images in each included study may have distinct features of *H pylori* infection and a clear and focused view, leading to a selection bias [28]. Second, the overfitting (modeling error that occurs when a certain learning model is excessively tailored to the training dataset and predictions are not well generalized to new datasets) of the AI algorithm cannot be excluded [31]. The diagnostic performance of the AI algorithms can only be valid for the population under evaluation and depends on the prevalence of target conditions for the selected population (so-called spectrum bias or class imbalance). The best and only way to prove the real performance of an AI algorithm is external (prospective) validation using unused datasets for model development, collected in a way that minimizes the spectrum bias [31]. However, there is no single study that adopted external validation for the performance of an established AI algorithm in this systematic review. Moreover, all the enrolled studies were conducted at a single center, which limits the generalization of the results. Third, there were little data regarding posteradication images, thus increasing the difficulty of the analysis of performance in the discrimination of uninfected and posteradicated images of *H pylori* infection. In real clinical practice, patients are not divided into only 2 categories of infected or noninfected patients. Indeed, there are many posteradicated patients, and this aspect should be reflected in the establishment of an AI algorithm. However, only 2 studies considered this category and conducted a separate analysis [9,10]. Because there were only 4 studies that conducted multiple tests in enrolling *H pylori*–infected patients, there may be a concern for selection bias. However, this factor is not expected to affect the overall results because there is a high probability of actual infection if any type of test is positive. Moreover, this factor was reflected in the methodological quality, and authors verified the effect of this bias through additional meta-regression. All the included studies were conducted in Asia, and no study confirmed the diagnostic validity of AI using external validation. Since the age of the enrolled population ranged from a mean of 48.6 years to a median of 64 years, excluding a younger population, further studies are required to understand the real value of the widespread use of this algorithm. Considering the high accuracy and real-time diagnostic characteristics, the results of this study indicate the clinical utility of using an AI algorithm as an additive tool for the prediction of *H pylori* infection during endoscopic procedures. It is highly likely that AI could replace endoscopists’ diagnoses of *H pylori* infections as guessed by visual inspection based on the evidence of this study. The real potential would be elucidated through the clinical application studies.

### Conclusion

In conclusion, an AI algorithm can be considered a reliable tool for endoscopic diagnosis of *H pylori* infection. The limitations of lacking external validation performance and being conducted only in Asia should be overcome.

## Appendix

Multimedia Appendix 1 Search strategy used to find relevant articles.

Multimedia Appendix 2 Data extraction, primary- and modifier-based analyses, and statistical analysis.

## Abbreviations

AI: artificial intelligence
AUC: area under the curve
BLI: blue-laser imaging
CNN: convolutional neural network
DOR: diagnostic odds ratio
FN: false negative
FP: false positive
IEE: image-enhanced endoscopy
LCI: linked color imaging
NBI: narrow-band imaging
NLR: negative likelihood ratio
NPV: negative predictive value
PLR: positive likelihood ratio
PRISMA-DTA: Preferred Reporting Items for a Systematic Review and Meta-analysis of Diagnostic Test Accuracy Studies
PROSPERO: International Prospective Register of Systematic Reviews
PPV: positive predicted value
QUADAS-2: Quality Assessment of Diagnostic Accuracy Studies–2
SROC: summary receiver operating characteristic
SVM: support vector machine
TN: true negative
TP: true positive
WLI: white-light imaging

## References

[1] Hooi JKY, Lai WY, Ng WK, Suen MMY, Underwood FE, Tanyingoh D, Malfertheiner P, Graham DY, Wong VWS, Wu JCY, Chan FKL, Sung JJY, Kaplan GG, Ng SC (2017). Global prevalence of Helicobacter pylori infection: systematic review and meta-analysis. Gastroenterology.

[2] Chey WD, Leontiadis GI, Howden CW, Moss SF (2017). ACG clinical guideline: treatment of Helicobacter pylori infection. Am J Gastroenterol.

[3] Bang CS, Lee JJ, Baik GH (2019). The most influential articles in Helicobacter pylori research: a bibliometric analysis. Helicobacter.

[4] Correa P (1988). A human model of gastric carcinogenesis. Cancer Res.

[5] (1994). IARC monographs on the evaluation of carcinogenic risks to humans. International Agency for Research on Cancer, ed Shistosomes, Liver Flukes and Helicobacter pylori. Vol. 61.

[6] (2014). IARC working group reports. International Agency for Research on Cancer, ed Helicobacter pylori Eradication as a Strategy for Preventing Gastric Cancer. Vol. 8.

[7] Bang CS, Baik GH, Shin IS, Kim JB, Suk KT, Yoon JH, Kim YS, Kim DJ (2015). Helicobacter pylori eradication for prevention of metachronous recurrence after endoscopic resection of early gastric cancer. J Korean Med Sci.

[8] Glover B, Teare J, Patel N (2020). A systematic review of the role of non-magnified endoscopy for the assessment of infection. Endosc Int Open.

[9] Shichijo S, Endo Y, Aoyama K, Takeuchi Y, Ozawa T, Takiyama H, Matsuo K, Fujishiro M, Ishihara S, Ishihara R, Tada T (2019). Application of convolutional neural networks for evaluating Helicobacter pylori infection status on the basis of endoscopic images. Scand J Gastroenterol.

[10] Yasuda T, Hiroyasu T, Hiwa S, Okada Y, Hayashi S, Nakahata Y, Yasuda Y, Omatsu T, Obora A, Kojima T, Ichikawa H, Yagi N (2020). Potential of automatic diagnosis system with linked color imaging for diagnosis of Helicobacter pylori infection. Dig Endosc.

[11] Dohi O, Majima A, Naito Y, Yoshida T, Ishida T, Azuma Y, Kitae H, Matsumura S, Mizuno N, Yoshida N, Kamada K, Itoh Y (2020). Can image-enhanced endoscopy improve the diagnosis of Kyoto classification of gastritis in the clinical setting?. Dig Endosc.

[12] Kim J (2018). Usefulness of narrow-band imaging in endoscopic submucosal dissection of the stomach. Clin Endosc.

[13] Cho B, Bang CS (2020). Artificial intelligence for the determination of a management strategy for diminutive colorectal polyps: hype, hope, or help. Am J Gastroenterol.

[14] Cho B, Bang CS, Park SW, Yang YJ, Seo SI, Lim H, Shin WG, Hong JT, Yoo YT, Hong SH, Choi JH, Lee JJ, Baik GH (2019). Automated classification of gastric neoplasms in endoscopic images using a convolutional neural network. Endoscopy.

[15] McInnes MDF, Moher D, Thombs BD, McGrath TA, Bossuyt PM, Clifford T, Cohen JF, Deeks JJ, Gatsonis C, Hooft L, Hunt HA, Hyde CJ, Korevaar DA, Leeflang MMG, Macaskill P, Reitsma JB, Rodin R, Rutjes AWS, Salameh J, Stevens A, Takwoingi Y, Tonelli M, Weeks L, Whiting P, Willis BH, PRISMA-DTA Group (2018). Preferred reporting items for a systematic review and meta-analysis of diagnostic test accuracy studies: the PRISMA-DTA statement. JAMA.

[16] Whiting PF, Rutjes AWS, Westwood ME, Mallett S, Deeks JJ, Reitsma JB, Leeflang MMG, Sterne JAC, Bossuyt PMM, QUADAS-2 (2011). QUADAS-2: a revised tool for the quality assessment of diagnostic accuracy studies. Ann Intern Med.

[17] Bang CS, Lee JJ, Baik GH (2019). Prediction of chronic atrophic gastritis and gastric neoplasms by serum pepsinogen assay: a systematic review and meta-analysis of diagnostic test accuracy. J Clin Med.

[18] Reitsma JB, Glas AS, Rutjes AWS, Scholten RJPM, Bossuyt PM, Zwinderman AH (2005). Bivariate analysis of sensitivity and specificity produces informative summary measures in diagnostic reviews. J Clin Epidemiol.

[19] Rutter CM, Gatsonis CA (2001). A hierarchical regression approach to meta-analysis of diagnostic test accuracy evaluations. Stat Med.

[20] Harbord RM, Whiting P (2018). Metandi: meta-analysis of diagnostic accuracy using hierarchical logistic regression. The Stata Journal.

[21] Zheng W, Zhang X, Kim JJ, Zhu X, Ye G, Ye B, Wang J, Luo S, Li J, Yu T, Liu J, Hu W, Si J (2019). High accuracy of convolutional neural network for evaluation of Helicobacter pylori infection based on endoscopic images: preliminary experience. Clin Transl Gastroenterol.

[22] Shichijo S, Nomura S, Aoyama K, Nishikawa Y, Miura M, Shinagawa T, Takiyama H, Tanimoto T, Ishihara S, Matsuo K, Tada T (2017). Application of convolutional neural networks in the diagnosis of Helicobacter pylori infection based on endoscopic images. EBioMedicine.

[23] Nakashima H, Kawahira H, Kawachi H, Sakaki N (2018). Artificial intelligence diagnosis of infection using blue laser imaging-bright and linked color imaging: a single-center prospective study. Ann Gastroenterol.

[24] Itoh T, Kawahira H, Nakashima H, Yata N (2018). Deep learning analyzes Helicobacter pylori infection by upper gastrointestinal endoscopy images. Endosc Int Open.

[25] Huang C, Chung P, Sheu B, Kuo H, Popper M (2008). Helicobacter pylori-related gastric histology classification using support-vector-machine-based feature selection. IEEE Trans Inf Technol Biomed.

[26] Huang C, Sheu B, Chung P, Yang H (2004). Computerized diagnosis of Helicobacter pylori infection and associated gastric inflammation from endoscopic images by refined feature selection using a neural network. Endoscopy.

[27] Best LM, Takwoingi Y, Siddique S, Selladurai A, Gandhi A, Low B, Yaghoobi M, Gurusamy KS (2018). Non-invasive diagnostic tests for Helicobacter pylori infection. Cochrane Database Syst Rev.

[28] Hoogenboom SA, Bagci U, Wallace MB (2020). Artificial intelligence in gastroenterology. The current state of play and the potential. How will it affect our practice and when?. Tech Innov Gastrointest Endosc.

[29] Abdullah R, Fakieh B (2020). Health care employees' perceptions of the use of artificial intelligence applications: survey study. J Med Internet Res.

[30] Tian Y, Liu X, Wang Z, Cao S, Liu Z, Ji Q, Li Z, Sun Y, Zhou X, Wang D, Zhou Y (2020). Concordance between Watson for oncology and a multidisciplinary clinical decision-making team for gastric cancer and the prognostic implications: retrospective study. J Med Internet Res.

[31] Yang YJ, Bang CS (2019). Application of artificial intelligence in gastroenterology. World J Gastroenterol.

[32] Bah A, Saraga E, Armstrong D, Vouillamoz D, Dorta G, Duroux P, Weber B, Froehlich F, Blum AL, Schnegg JF (1995). Endoscopic features of Helicobacter pylori-related gastritis. Endoscopy.

[33] Laine L, Cohen H, Sloane R, Marin-Sorensen M, Weinstein WM (1995). Interobserver agreement and predictive value of endoscopic findings for H. pylori and gastritis in normal volunteers. Gastrointest Endosc.

[34] Redéen S, Petersson F, Jönsson K, Borch K (2003). Relationship of gastroscopic features to histological findings in gastritis and Helicobacter pylori infection in a general population sample. Endoscopy.

[35] Dohi O, Yagi N, Onozawa Y, Kimura-Tsuchiya R, Majima A, Kitaichi T, Horii Y, Suzuki K, Tomie A, Okayama T, Yoshida N, Kamada K, Katada K, Uchiyama K, Ishikawa T, Takagi T, Handa O, Konishi H, Naito Y, Itoh Y (2016). Linked color imaging improves endoscopic diagnosis of active Helicobacter pylori infection. Endosc Int Open.

[36] Takeda T, Asaoka D, Nojiri S, Nishiyama M, Ikeda A, Yatagai N, Ishizuka K, Hiromoto T, Okubo S, Suzuki M, Nakajima A, Nakatsu Y, Komori H, Akazawa Y, Nakagawa Y, Izumi K, Matsumoto K, Ueyama H, Sasaki H, Shimada Y, Matsumoto K, Osada T, Hojo M, Kato M, Nagahara A (2019). Linked color imaging and the Kyoto classification of gastritis: evaluation of visibility and inter-rater reliability. Digestion.

[37] Yagi K, Saka A, Nozawa Y, Nakamura A (2014). Prediction of Helicobacter pylori status by conventional endoscopy, narrow-band imaging magnifying endoscopy in stomach after endoscopic resection of gastric cancer. Helicobacter.

[38] Kanzaki H, Uedo N, Ishihara R, Nagai K, Matsui F, Ohta T, Hanafusa M, Hanaoka N, Takeuchi Y, Higashino K, Iishi H, Tomita Y, Tatsuta M, Yamamoto K (2012). Comprehensive investigation of areae gastricae pattern in gastric corpus using magnifying narrow band imaging endoscopy in patients with chronic atrophic fundic gastritis. Helicobacter.

