# Supplementary material for: Artificial Intelligence for the Prediction of Helicobacter Pylori Infection in Endoscopic Images: Systematic Review and Meta-Analysis Of Diagnostic Test Accuracy
Source: J Med Internet Res. 2020 Sep 16;22(9):e21983. doi: 10.2196/21983 (PMC7527948; doi:10.2196/21983)
Supplement: Multimedia Appendix 1 [file jmir_v22i9e21983_app1.docx]

**Multimedia Appendix 1. Searching strategy to find the relevant articles.**

| **Database: MEDLINE (through PubMed)** |
| --- |
| #1 "artificial intelligence"[tiab] OR "AI"[tiab] OR "deep learning"[tiab] OR "machine learning"[tiab] OR "computer aided"[tiab] OR "neural network"[tiab] OR "CNN"[tiab]: 102697  #2 "Helicobacter pylori"[tiab] OR “Helicobacter”[Mesh]: 45043  #3 #1 AND #2: 94  #4 #3 AND English[Lang]: 89 |
| **Database: Embase** |
| #1 'artificial intelligence':ab,ti,kw OR 'AI':ab,ti,kw OR 'deep learning':ab,ti,kw OR 'machine learing':ab,ti,kw OR 'computer aided':ab,ti,kw OR 'neural netwokr':ab,ti,kw OR 'CNN':ab,ti,kw: 75482  #2 'Helicobacter pylori':ab,ti,kw OR 'Helicobacter'/exp: 68419  #3 #1 AND #2: 102  #4 #3 AND ([article]/lim OR [article in press]/lim OR [review]/lim) AND [English]/lim: 68 |
| **Database: Cochrane Library** |
| #1 artificial intelligence:ab,ti,kw or AI:ab,ti,kw or deep learning:ab,ti,kw or machine learning:ab,ti,kw or computer aided:ab,ti,kw or neural network:ab,ti,kw: 6967  #2 MeSH descriptor: [Helicobacter] explode all trees: 1949  #3 #1 AND #2: 4 |
