# Supplementary material for: Artificial Intelligence for the Prediction of Helicobacter Pylori Infection in Endoscopic Images: Systematic Review and Meta-Analysis Of Diagnostic Test Accuracy
Source: J Med Internet Res. 2020 Sep 16;22(9):e21983. doi: 10.2196/21983 (PMC7527948; doi:10.2196/21983)
Supplement: Multimedia Appendix 2 [file jmir_v22i9e21983_app2.docx]

**Multimedia Appendix 2**

**Data extraction and primary and modifier-based analyses.**

Two evaluators (C.S.B. and J.J.L.) independently used the same data form to collect the summary of primary outcomes (TP, FP, FN, and TN) and modifiers in each study. For the studies with incomplete data, we contacted the corresponding author of each study by e-mail to obtain the exact values of the following primary outcomes: TP, FP, TN, and FN.

The primary outcome of this study was diagnostic test accuracy. We calculated the values of TP (subjects with a positive finding using AI who have *H. pylori* infection on endoscopic images), FP (subjects with a positive finding using AI who do not have *H. pylori* infection on endoscopic images), FN (subjects with a negative finding using AI who have *H. pylori* infection on endoscopic images), and TN (subjects with a negative finding using AI who do not have *H. pylori* infection on endoscopic images) using AI for the prediction of *H. pylori* infection in each study. To calculate the values, we applied 2 × 2 tables whenever possible to the data of the original articles that contained various diagnostic performance indices (sensitivity, specificity, PPV, NPV, PLR, NLR, accuracy, or DOR etc.). If only a portion of the data was presented, we calculated the values for TP, FP, FN, and TN using the following formulas: sensitivity = TP/(TP+FN); specificity = TN/(FP+TN); PPV = TP/(TP+FP); NPV = TN/(FN+TN); PLR = sensitivity/(1-specificity); NLR = (1-sensitivity)/specificity; accuracy = (TP+TN)/(TP+FP+FN+TN); DOR = (TP × TN)/(FP × FN); standard error = (ln(upper confidence interval (CI)) – ln(lower CI))/3.92 = √(1/TP+1/FP+1/FN+1/TN) [17].

The following data were also extracted from each study whenever possible: study design, age, sex, sample size, published year, type of AI for the establishment of the algorithm, and type of endoscopic images, such as WLI, LCI, or BLI.

Statistical analysis

Stata Statistical Software versions 15.1 (College Station, Texas, US), including relevant packages of metandi and midas were used for the meta-analysis. A descriptive synthesis was conducted through systematic review, and quantitative synthesis [bivariate random model [18] and hierarchical summary receiver operating characteristic (HSROC) model [19]] was performed if the included articles were sufficiently homogenous. The common effect size (TP, FP, FN, and TN) was extracted or calculated for each study. A Forest plot of pooled sensitivity or specificity using a bivariate model, as well as a summary receiver operating characteristic (SROC) curve using a HSROC model, were generated and presented. Heterogeneity across the studies was determined by the correlation coefficient between logit transformed sensitivity and specificity using a bivariate model [18] and asymmetry parameter, β (beta), where β=0 corresponds to a symmetric receiver operating characteristic (ROC) curve, in which the DOR does not vary along the curve according to the HSROC model [19,20]. A positive correlation coefficient (greater than 0), and β with significant *P* value (*P* <.05), indicates heterogeneity between the studies [19,20]. Visual examination of the SROC curve was also performed to find heterogeneity. Subgroup analysis and univariable meta-regression using the modifiers identified during the systematic review were also conducted to confirm the robustness of the main result and to identify the reasons for heterogeneity. Bivariate and HSROC models have been proposed to overcome the limitation (does not consider the heterogeneity between studies) of the previous used ‘Moses-Shapiro-Littenberg method’. However, the metandi and midas package of STATA require a minimum of four studies to conduct the diagnostic test accuracy meta-analysis. It was impossible to conduct the subgroup analysis in the image-based studies. Therefore if less than four studies are enrolled in the subgroup analysis, Meta-DiSc 1.4 (XI Cochrane Colloquium, Barcelona, Spain) using the ‘Moses-Shapiro-Littenberg method’ was used. Publication bias was evaluated using the Deeks’ funnel plot asymmetry test.
